# Validation of the Amsterdam Dynamic Facial Expression Set – Bath Intensity Variations (ADFES-BIV): A Set of Videos Expressing Low, Intermediate, and High Intensity Emotions

**DOI:** 10.1371/journal.pone.0147112

**Published:** 2016-01-19

**Authors:** Tanja S. H. Wingenbach, Chris Ashwin, Mark Brosnan

**Affiliations:** Department of Psychology, University of Bath, Bath, United Kingdom; University of Udine, ITALY

## Abstract

Most of the existing sets of facial expressions of emotion contain static photographs. While increasing demand for stimuli with enhanced ecological validity in facial emotion recognition research has led to the development of video stimuli, these typically involve full-blown (apex) expressions. However, variations of intensity in emotional facial expressions occur in real life social interactions, with low intensity expressions of emotions frequently occurring. The current study therefore developed and validated a set of video stimuli portraying three levels of intensity of emotional expressions, from low to high intensity. The videos were adapted from the Amsterdam Dynamic Facial Expression Set (ADFES) and termed the Bath Intensity Variations (ADFES-BIV). A healthy sample of 92 people recruited from the University of Bath community (41 male, 51 female) completed a facial emotion recognition task including expressions of 6 basic emotions (anger, happiness, disgust, fear, surprise, sadness) and 3 complex emotions (contempt, embarrassment, pride) that were expressed at three different intensities of expression and neutral. Accuracy scores (raw and unbiased (*Hu*) hit rates) were calculated, as well as response times. Accuracy rates above chance level of responding were found for all emotion categories, producing an overall raw hit rate of 69% for the ADFES-BIV. The three intensity levels were validated as distinct categories, with higher accuracies and faster responses to high intensity expressions than intermediate intensity expressions, which had higher accuracies and faster responses than low intensity expressions. To further validate the intensities, a second study with standardised display times was conducted replicating this pattern. The ADFES-BIV has greater ecological validity than many other emotion stimulus sets and allows for versatile applications in emotion research. It can be retrieved free of charge for research purposes from the corresponding author.

## Introduction

Humans are very social, so it is not surprising our attention is often drawn to faces (e.g. [1]). This can be explained by the multitude of information faces convey about others, which includes such invariant aspects as their sex [2], race [3], and age [4]. We also perceive dynamic features about others from the face, including facial expressions. Facial emotional expressions are utilised within social interactions because they serve several functions. Darwin [5] proposed that facial expressions of emotion are directly linked to the feeling of an emotion, so that facial expressions provide a visual display of the internal emotional states of others. Since these signals about emotional states can be interpreted by an observer, emotional expressions serve a communicative role [6]. Facial expressions of emotion can also be used to regulate the environment, by indicating people’s intentions and actions [7]. When used in a more functional way for social regulation, expressions do not necessarily have to accurately reflect the current emotional state [6]. Given the importance of facial emotional expressions for social interactions and for conveying crucial information about others and ourselves [8], they have attracted a vast amount of research investigating our ability to correctly interpret those expressions.

To date, most facial emotion recognition research has utilised static stimuli with a high intensity of the facial expressions [9–12]. This includes the “Pictures of Facial Affect” [13], which might be the most widely used standardised face emotion stimulus set for research [14]. Based on the Pictures of Facial Affect, the “Facial Action Coding System” (FACS) was developed, where all muscular movements underlying facial expressions are catalogued [15]. These facial muscle movements are called ‘action units’ and specific combinations of such action units have been attributed to specific prototypical emotional facial expressions. For example, a pattern of muscle movements in the face with the lip corners pulled up and crow’s feeds at the outer edges of the eyes corresponds to ‘happiness’ [16], which is known as the Duchenne smile [17]. The development and validation of facial emotion expression stimulus sets often involves coding according to the FACS (e.g. [18]).

Research using static stimuli has helped identify the ‘basic emotions’, which are the facial expressions thought to be distinct and recognisable by all humans independent of their culture or race [19, 20, 21], although this view has been questioned by some (e.g. [22, 23]). The six basic emotions consist of anger, disgust, fear, happiness, sadness, and surprise [19]. These emotions are usually well recognised in experiments, although the recognition rates generally vary between the emotions [24]. The high recognition rate of basic emotions has adaptive functions, since it allows for rapid responses to biologically relevant stimuli [19]. The emotions shown to be easiest to recognise throughout the literature are happiness and surprise (e.g. [25, 26]), with fear often being the hardest to recognise (e.g. [27]). Not only accuracy, but also response times are influenced by item difficulty. For facial emotion recognition that means that response times are shortest for clear and unambiguous, easy to recognise, facial expressions (e.g. [28]). Accordingly, facial expressions of happiness are faster recognised than negative emotions [29]. Nonbasic emotions are assumed to have their basis in basic emotions [30], but are paired with self-evaluations [31] and are more likely to be influenced by culture [32]; examples constitute pride and embarrassment, which are called *complex* emotions. Complex emotions are generally less well recognised than basic emotions within facial emotion recognition experiments (e.g. see [33]).

Thanks to technological advancements it has become possible to conduct research on facial expressions using dynamic stimuli, which are more aligned to the real-life emotional expressions that are being studied. The application of dynamic stimuli poses the advantage of increased ecological validity. Static images only capture one moment in time, while dynamic stimuli enable the display of the whole progression from neutral to the full apex of the facial expression of emotion. Static images only display the activated facial action units constructing the facial emotional expression, whereas dynamic stimuli provide additional cues, such as temporal characteristic of the activation of the facial features, which are used in decoding of facial expressions [9, 12, 34]. In line with that, it has been suggested that in addition to static characteristics also dynamic characteristics are embedded in our representations of emotional facial expressions [35, 36]. Accordingly, facial emotion recognition research has shown that dynamic stimuli lead to higher recognition rates than static stimuli (e.g. [12, 22, 37]).

There are two types of dynamic face stimuli, video sequences based on morphed images and true video recordings of real human faces. Morphed dynamic stimuli are created by morphing two original static images, which may include images of a neutral face and an emotional expression, gradually into each other by creating artificial images according to predefined linear increments (e.g. [35, 38, 39]). The morphing technique allows for a high level of standardisation, as the number of increments and therewith the number of images (frames) as well as the presentation time of each frame and thereby the exposure time can be kept constant across all sequences. Morphed sequences are especially useful when investigating sensitivity in emotion perception from faces (e.g. [40–42]). However, the forced simultaneous changes of facial features that come with morphing pose a limitation for application in facial emotion recognition experiments. The naturalness of computer-generated expressions is questionable, as it is unclear whether the created movements are anatomically feasible [36, 43]. This concerns the onsets of single facial action units, which can vary [44], and the speed of those action units in reaching apex, which varies between emotions (Hara and Kobayashi as cited by [45]). Conversely, true video recordings preserve and capture variations in onsets and speed of facial action units. This has sparked the development of video recordings where professional actors or untrained participants are filmed whilst displaying prototypical facial emotional expressions (e.g. the Amsterdam Dynamic Facial Expression Set, ADFES [33]; Geneva Multimodal Emotion Portrayals, GEMEP [46]; Multimodal Emotion Recognition Test, MERT [47]; [48]; the MPI Facial Expression Database [49]).

One important feature not typically included in published face emotion stimulus sets is variations in intensity level of expressions. Including varying intensities is important, as in social interactions the facial expressions that are displayed spontaneously are mostly of low to intermediate intensity [50] with full intensity expressions being the exception [51]. Subtle displays of face emotion are very commonly seen and therefore are a major part of facial emotion recognition [43]. It has been proposed that people generally are not overly good at recognising subtle expressions [52], and research from static morphed images of varying intensities showed that accuracy [44] and response time [53] are linearly associated with physical expression intensity. Ekman and Friesen [15] suggested intensity ratings in the FACS from trace to maximum highlighting the importance of considering the whole range of emotional expression intensity. Including subtle expressions allows for a broader and more reliable assessment of facial emotion recognition.

Moreover, face emotion stimuli of varying intensities of facial expressions allow for investigation of populations that are thought to have difficulties with facial emotion recognition (e.g. in Autism Spectrum Disorders; for a review see [54]), where it can be examined whether those difficulties present across all intensity levels or for example just for subtler displays. Performance in facial emotion recognition at varying intensities is not only of interest for clinical samples, but also for general populations. For example, a female advantage compared to males in facial emotion recognition is frequently reported (e.g. [55]), however, this is mostly based on full intensity and/or static expressions. A potential research question to investigate is whether females are consistently better than males at recognising facial emotional expressions or whether the advantage is more prominent at certain intensities. Together, stimuli of varying intensities of facial emotional expressions have the advantage to allow for a more specific investigation of group differences in facial emotion recognition or emotion perception.

To our knowledge, there are only a very limited number of stimulus sets including varying intensity of emotional expressions based on dynamic stimuli. One stimulus set containing computer-morphed videos for the six basic emotions at varying intensities based on morphing neutral and emotional expressions has been published (the Emotion Recognition Task, ERT; [40]) and two true video stimulus sets including varying intensities; the Database of Facial Expressions, DaFEx [56]; and the MPI Facial Expression Database [49]. The DaFEx [56] includes three intensity levels of expression for the six basic emotions, however, is limited in the use for emotion recognition research from faces, as emotions are expressed not only facially but also with body posture providing further cues useful for decoding. Additionally, the stimuli vary in length by up to 20 seconds. The MPI contains 55 facial expressions of cognitive states (e.g. remembering) and five basic emotions with context variations at two intensity levels each. However, in the MPI the people expressing the emotions facially (encoders) wear a black hat with several green lights on for head tracking purposes which also covers facial features generally visible in social interactions (e.g. hair)–only the face is visible—all of which lowers ecological validity. Validation data have only been published for the high intensity expressions, not low intensity. None of the two sets of facial emotion expressions have been standardised by FACS-coding. To date, there is no published and validated stimulus set containing video recordings of facial emotional expressions including varying intensities of expressions of the six basic and also complex emotions. It is advised to include a wide range of emotions in order to increase ecological validity.

The present research searched for a video stimulus set of basic and complex emotional expressions and applied the following criteria to increase ecological validity: 1) the stimuli need to be in colour, 2) standardisation of the encoders’ emotional expressions based on FACS by certified coders, 3) having the whole head, but not the rest of the body of the encoder visible in the videos, 4) have no utterances included to avoid distraction and unwanted further cues to the emotion, 5) a large number of encoders per emotion included, and 6) containing a wide range of emotional expressions.

The ADFES [33] was identified to meet the criteria outlined above. The ADFES is comprised of 12 Northern European encoders (7 male, 5 female) and 10 Mediterranean encoders (5 male, 5 female) expressing six basic emotions and three complex emotions facially: contempt, pride, embarrassment, as well as neutral expressions. The videos are all 5.5 seconds in length, and there are versions of each video with encoders facing direct into the camera and also from a 45° angle. It is a clear advantage of the ADFES that it contains facial expressions of not only the six basic but also three complex emotions (next to neutral), especially for application in facial emotion recognition research. A low number of emotions may not accurately assess emotion recognition. It has been suggested that a low number of possible emotion response alternatives could constitute a discrimination task, since the participant is asked to discriminate between low numbers of options, which can be mastered by applying exclusion criteria at the cost of the results’ validity [57]. For example, if only one positively valenced emotion is included (usually happiness), then the simple discrimination between positive and negative can lead to full accuracy for happiness rather than actually recognising happiness [58]. However, if the six standard basic emotions and complex emotions (e.g. pride), are included, that increases the likelihood of having more than one positively valenced emotion included in the task and thereby demand more recognition than discrimination ability. A wider range of emotions included in a stimulus set therefore not only aids ecological validity, also the results’ validity. However, since the ADFES videos only display high intensities of emotional expressions at their endpoint, the current research aimed to create and validate an expanded standard video stimulus set that includes both high intensity and more subtle expressions on the basis of the Northern European and face forward videos from the ADFES; the Amsterdam Dynamic Facial Expression Set—Bath Intensity Variations (ADFES-BIV).

The videos were edited to display three levels of intensity of facial emotional expression: low, intermediate, and high. The general aim of the current study was to test the validity of the ADFES-BIV, with results showing this new video set has good validity in order to be a useful tool in emotion research. It was aimed to validate these intensity levels as distinct categories on the basis of their accuracies by replicating the pattern from static images, i.e. low intensity expressions would have lower accuracy rates than the intermediate intensity expressions, which themselves would have lower accuracy rates than the high intensity expressions. Furthermore, it was expected that the intensity levels would differ on response latencies with the same pattern of effect as the accuracy rates with fastest responses to high intensity expressions, as they portray the emotions most clearly.

It was further aimed to test the ADFES-BIV’s emotion categories and the emotions at each intensity level for validity on the basis of raw hit rates as well as unbiased hit rates (*Hu*; [59]). It was expected that all categories would have recognition rates significantly above chance level on raw and unbiased hit rates. It was expected that the recognition rates would vary between emotions and the influence of expression intensity was investigated. That is, are emotions that are easy to recognise at high intensity (i.e. surprise, happiness) also easy to recognise at low intensity with accuracies comparable to the accuracies at high intensity? It was further expected that the emotions with highest recognition would also be the emotions with fastest responses and the emotions with lowest recognition the ones with slowest responses.

## Study 1

### Method

#### Participants

The sample consisted of 92 adult participants (41 male, 51 female) recruited from the University of Bath community. Participants were aged between 18 and 45 (*M* = 23.25, *SD* = 5.65) and all had normal or corrected-to-normal vision. Participants were enrolled as Undergraduate students (*n* = 54), Masters students (*n* = 8), PhD students (*n* = 22), and staff at the University (*n* = 8). Undergraduate participants from the Department of Psychology at the University of Bath gained course credit for participation, while all other participants were compensated with £5 for participation. None of the participants reported a clinical diagnosis of a mental disorder. The data on sex differences based on the ADFES-BIV will be reported elsewhere.

#### Stimuli development

Permission to adapt the videos was obtained from one of the authors of the ADFES (Fischer A. Personal communication. 19 April 2013). The 10 expressions included in the present research were anger, contempt, disgust, embarrassment, fear, happiness, neutral, pride, sadness, and surprise. Each of the emotions was expressed by 12 encoders; 7 male and 5 female. For each of the 120 videos from the Northern European set (12 encoders x 10 expressions) three new videos displaying three different stages of expression: low, high, and intermediate were created by extracting consecutive frame sequences starting with a neutral frame (i.e. blank stare). From the neutral expression videos, three different sequences were extracted as well to obtain an equal number of videos per category. Additionally, 10 videos were created for one encoder from the Mediterranean set of the ADFES [33] to be used as an example display of each emotional expression included in the set. This led to a total of 370 videos. The length of each of the videos was set to 26 frames with a frame rate of 25/sec, consistent with the original ADFES [33]. The resulting videos were all 1040ms in length. Since apex of facial expression is generally reached within 1 second for basic emotions (Hara & Kobayashi as cited by [45]), this timing allowed for all videos to start with a neutral facial expression and to continue until the end of the expression. This is more ecologically valid, since outside the laboratory people get to see neutral expressions as a point of reference [43].

**Fig 1:**
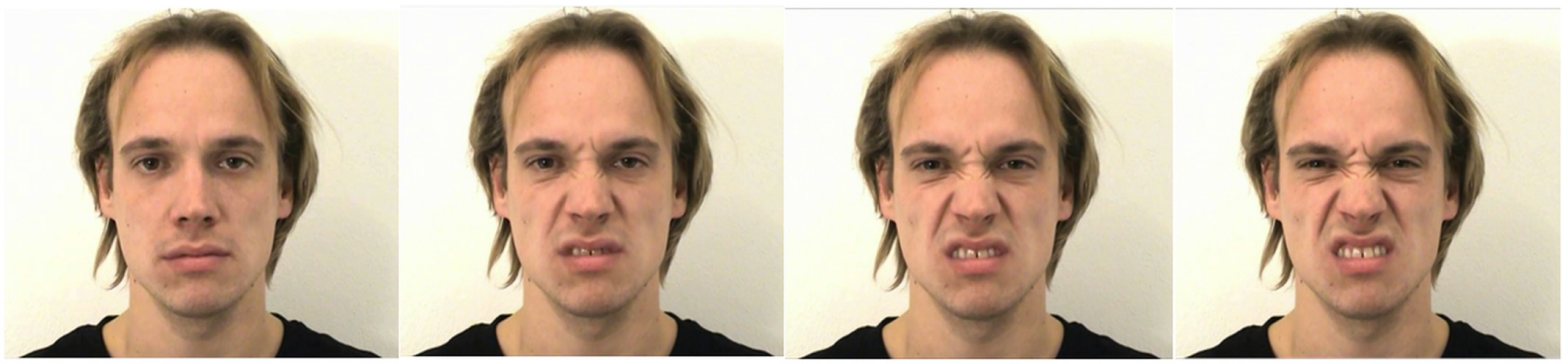
Neutral expression, last frame of disgust at low, intermediate, and high expression intensity (from left to right).

The three intensity levels created with the current research were defined by adopting the operationalization of Bould and Morris [34]. Accordingly, subtle expressions of face emotion were more ambiguous in nature [51], whereas the high intensity expressions were generally more unambiguous. A similar approach to create the varying intensities of facial emotional expression was followed as was carried out in Bould and Morris [34], where the unfolding facial emotional expressions, from neutral expression to full intensity, were truncated. Precisely, for each new video, the desired frame for each stage of expression corresponding to the appropriate intensity level (e.g. low, intermediate, and high) was selected, and then 25 consecutive frames were included prior to that frame. This created videos of equal length but with the last frame corresponding to the relevant level of emotional intensity. The different intensities reflected the spatial distance of the facial features in comparison to their location within the neutral expression, based on the degree of contraction of the relevant facial muscles for that emotional category. The category of ‘low’ intensity included subtle expressions where only limited degrees of action units in the face are visible. The ‘high’ intensity category included the apex of the emotional expressions within the videos, which involved the maximal contraction of all the relevant facial action units and the greatest spatial distance of facial features from where they appeared in the neutral expression. The ‘intermediate’ category was chosen to be as distinct as possible from the low and high intensity points in the videos, with the muscle contraction and movement of the facial features being mid-point between those intensities (see Fig 1). The individual in this manuscript has given written informed consent (as outlined in PLOS consent form) to publish his photograph. The categorisation of the videos as being low, intermediate, or high intensity was subsequently confirmed with a study asking participants (*N* = 30; 50:50 sex ratio) to judge the intensity of the facial expressions on a visual analogue scale ranging from very low to very high intensity (respective to 0–100%). Ethical approval for this study was given by the University of Bath Psychology Ethics Committee and all participants gave written informed consent prior to participation. On average, the perceived intensity of the low intensity videos was 42% (*SD* = 9.80), 55% for the intermediate intensity videos (*SD* = 8.54), and the perceived mean intensity of the high intensity videos was 66% (*SD* = 7.05), whereas the neutral faces were rated with 9% on average (*SD* = 9.88). The differences between the categories were statistically significant as identified by paired sample *t*-tests (neutral-low: *t*(29) = -15.14, *p* < .001; low-intermediate: *t*(29) = -16.62, *p* < .001; intermediate-high: *t*(29) = -21.23, *p* < .001). The linear increase of perceived intensity with increasing intensity level is in line with reports from previous research (e.g. [60]).

#### Facial emotion recognition task

The psychological stimuli presentation software E-Prime 2.0 [61] was used to display the emotion recognition experiment and record responses. The participants began the experiment with an affective state assessment on a 5-point Likert scale rating valence and arousal using the non-verbal Self-Assessment Manikin (SAM; [62]) before and after watching a neutral clip; a short documentary about fertilisers (4 minutes and 18 seconds). This clip aimed to settle the participants for the experiment in case any strong and distracting feelings might have been present. The ratings on valence (before: *M* = 3.76, *SD* = 0.75, after: *M* = 3.41, *SD* = 0.71, *Z* = -4.33, *p* < .001) and arousal (before: *M* = 1.93, *SD* = 0.82, after: *M* = 1.78, *SD* = 0.81, *Z* = -1.99, *p* = .047) changed significantly from before to after the neutral clip, so that afterwards the mood of participants was ‘neutral’. Participants then completed 10 practice trials of the emotion recognition task, which included examples of all 10 emotional expressions of one encoder from the Mediterranean set of the ADFES [33] to familiarise participants in general with the task procedures. The 10 example videos of the Mediterranean encoder did not appear again in the experiment; only the Northern European set was used for validation. The answer screen was presented to participants before the practice started to familiarise them with the answer categories, and to ensure they understood all emotion terms. If participants did not understand a category, then definitions were provided from the Oxford English Dictionary. The answer screen contained a list of all the emotion category choices of anger, contempt, disgust, embarrassment, fear, happiness, neutral, pride, sadness, surprise. The answer choices were equally distributed over the screen in 2 columns and 5 rows and appeared in alphabetical order. The answer screen always appeared the same way throughout the experiment to avoid participants having to search for a term and thereby biasing response times. The mouse position was not fixed which allowed for proximity to answer categories to differ between trials. Participants used the mouse to click on their chosen answer on the screen when they made their decision, and were asked to respond immediately. Instruction was given to have their hand always on the mouse, so that when the answer slide appeared they could click immediately. The experiment consisted of 360 trials (12 encoders x 10 expressions x 3 intensities) presented in random order for each participant.

Each trial started with a fixation cross for 500ms prior to the presentation of each emotion video. After each video a blank slide appeared for 500ms followed by the answer screen. An infinite response time was chosen to avoid restricting participants in their answer time producing trials with no response. An accidental mouse click outside the valid answer choice fields on the response screen prompted it to re-appear to further avoid missing responses. No feedback about their answer was provided. The mouse cursor only appeared for the emotion labelling display within trials. This way, it could not serve as distraction from the video on the screen. The resolution for the experiment was set to 1024 x 768 on a 21-inch monitor and participants were seated approximately 60cm from the computer screen, which allowed the face stimuli to appear in approximately full-size (1024 x 768) to the participant similar to face-to-face social interactions outside the laboratory.

#### Questionnaires

Two different questionnaires were included: the Symptom-Checklist 90-R (SCL-90-R; [63]) and the Autism Spectrum Quotient (AQ; [64]). The AQ data was not used in the analyses here and will be presented elsewhere. The SCL-90-R served as screening instrument for potential clinical disorders since a healthy sample was desired. The Global Severity Index (GSI), i.e. the SCL-90-R sum score, was used as a distress index and marker for potential caseness according to the developer’s suggestion of a GSI score equivalent to a T score of 63. Twenty-six participants scored outside the normal range for non-patients. To test whether or not those individuals influenced the overall accuracy of response, analyses were run with and without these participants included. Only minor changes of less than 1% resulted (total accuracy without ‘cases’ = 69.47% vs. with ‘cases’ = 68.81%); a 1-sample *t*-test did not identify the difference as significant (*p* > .05). Therefore, results are presented including those participants.

#### Procedure

The testing session was conducted in a quiet laboratory at the University of Bath, with between one and of four participants tested simultaneously. All participants underwent the computer task followed by the questionnaires with participants wearing headphones throughout the testing. Participants were fully debriefed on completion of the study and compensated for participation or granted course credit where applicable. Ethical approval for this study was given by the University of Bath Psychology Ethics Committee and all participants gave written informed consent prior to participation.

### Dependent variables

DV 1: *Raw hit rates* referred to the percentage correct out of the total number of trials for each category. Since there were 10 answer choices on each trial, the chance level of response was 10%. Recognition rates above 10% were therefore necessary for a category to be considered recognisable at a greater than chance level of responding. No data were excluded.

DV 2: *Unbiased hit rates* (*Hu*) were calculated based on the formula provided by Wagner [59] for each *emotion* category and each *emotion* category across all *intensity* levels. The formula corrects the accuracy rates by the possibility of choosing the right emotion label by chance as well as answering habits and produces so called ‘unbiased hit rates’, which additionally have the advantage of making the results comparable across studies. In facial emotion recognition tasks where multiple answer choices are provided, there is the possibility for the participant to choose the correct emotion label by chance, which biases the accuracy rates (percentage correct out of all presentations for a category). In addition, answering habits can occur, which also bias the results. An extreme example for such an answering habit would be that a participant assigns one specific emotion category to any sort of emotional display, e.g. always surprise for all fear and surprise displays. This would result in a perfect score for surprise, but does not reflect the ability to recognise surprise, as all fear presentations would be misattributed as surprise. To account for those potential biases, Elfenbein and Ambady [65] advised to use the formula proposed by Wagner [59] for multiple choice facial emotion recognition tasks. No data were excluded.

DV 3: *Response time* referred to the time participants took to respond from the moment the answer screen was presented until the participant clicked the mouse on their answer choice. Mean response times were computed for each *intensity* level and *emotion* category. Only trials with correct responses were used in these analyses.

### Results

#### DV 1: Raw hit rates

The overall accuracy for the task was 69% (*SD* = 9.02). Taken together, all low intensity videos had a mean raw hit rate of 56% (*SD* = 11.11), 68% for intermediate intensity (*SD* = 10.51), and 75% for high intensity (*SD* = 9.94).

**Fig 2:**
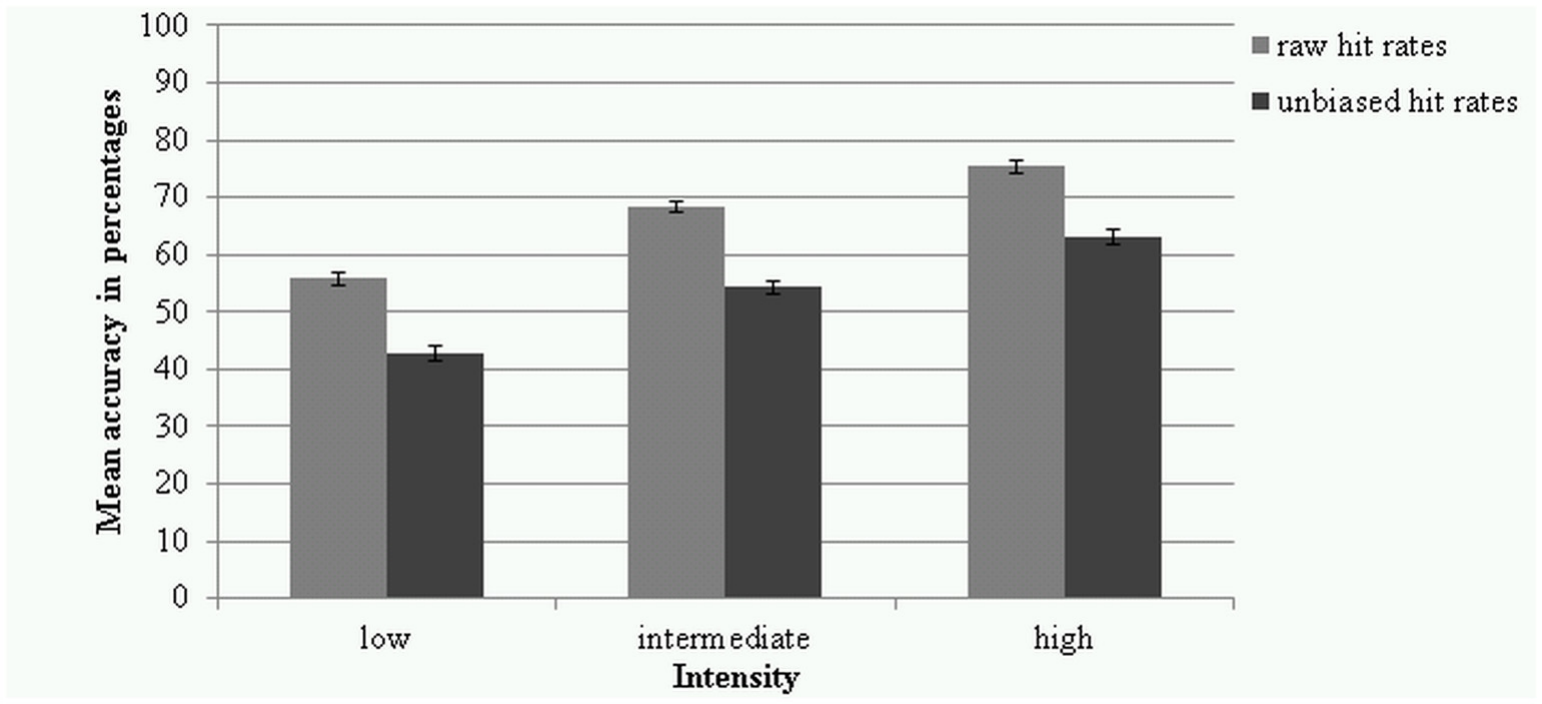
Raw and unbiased hit rates in percentages for the 3 intensity levels. Error bars represent standard errors of the means.

Most of the emotion categories were non-normally distributed with some left- and some right-skewed according to the histograms, and transformations did not normalise the data. Due to the robustness to normality violations [66,67], repeated measures ANOVA was conducted. A 3 (intensities) x 9 (emotions) repeated measures ANOVA with Greenhouse-Geisser adjustment of degrees of freedom was applied due to violation of Sphericity. Neutral was excluded from this analysis, since it does not have varying intensities of expression. There was a significant main effect of *intensity* (*F*(1.72, 156.62) = 491.80, *p* < .001, partial *η²* = .844, power = 1.000) and pairwise comparisons showed the intensity levels were all significantly different from each other (*p*’s < .001) (see Fig 2).

**Fig 3:**
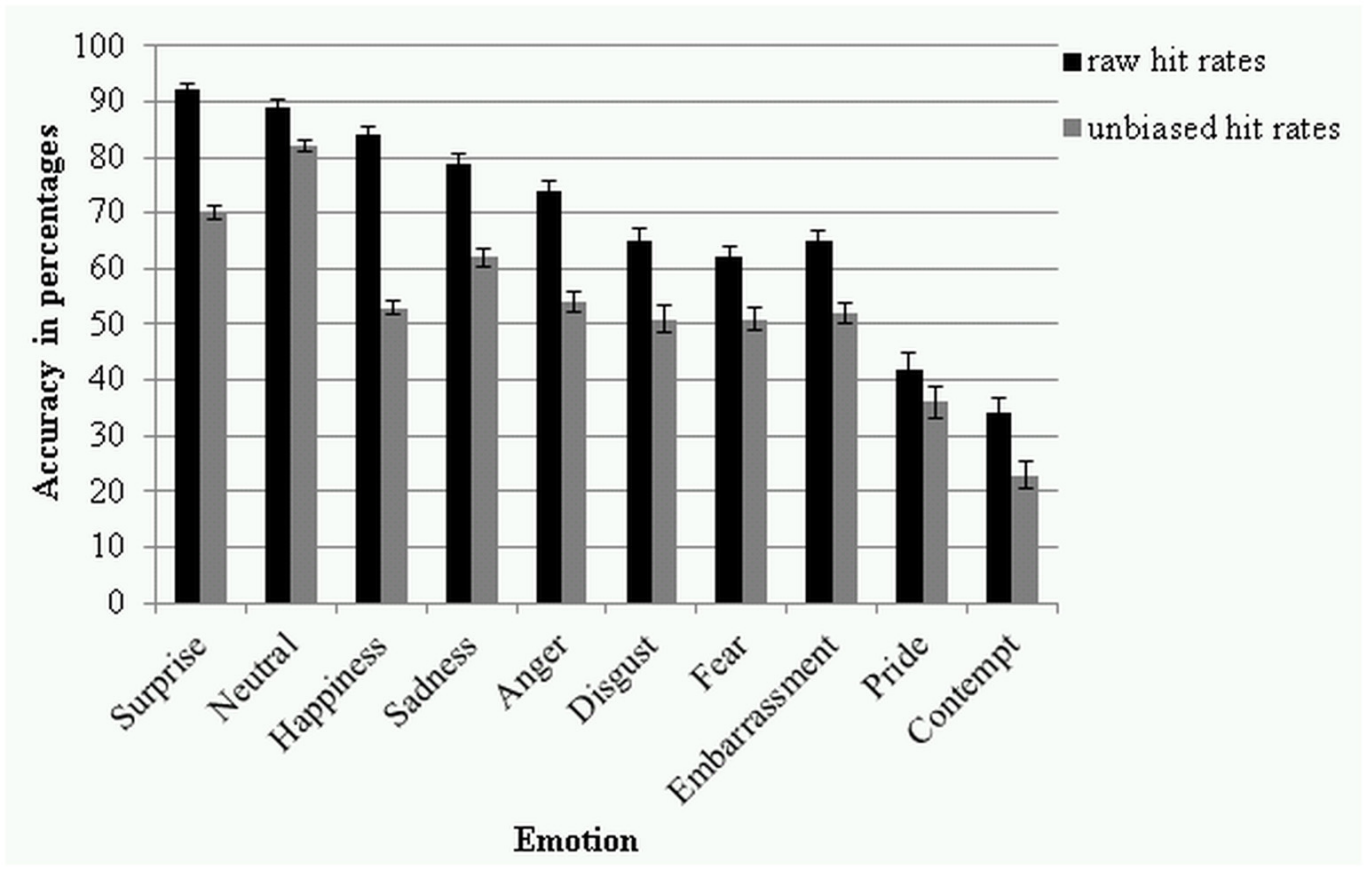
Raw and unbiased hit rates in percentages for the 10 emotion categories. Error bars represent standard errors of the means.

The main effect of *emotion* was significant (*F*(5.72, 520.43) = 94.81, *p* < .001, partial *η²* = .510, power = 1.000) (see Fig 3). Pairwise comparisons showed that the raw hit rates of the emotion categories were significantly different from each other (*p*’s < .028) with only a few exceptions; disgust and embarrassment did not differ significantly from each other (*p* = .856), as so disgust and fear (*p* = .262), and embarrassment and fear (*p* = .281). The means and standard deviations of the raw hit rates for the 9 *emotion* categories and neutral are presented in Table 1.

**Table 1:** Raw Hit Rates (*H*) and Unbiased Hit Rates (*Hu*) for the 10 Emotion Categories.

Emotion (*n* = 92)	*H*		*Hu*	
	*M* %	*SD* %	*M* %	*SD* %
**Anger**	74%	18.56	54%	18.26
**Contempt**	34%	24.28	23%	24.07
**Disgust**	65%	22.69	51%	23.44
**Embarrassment**	65%	17.44	52%	18.67
**Fear**	62%	20.42	51%	20.53
**Happiness**	84%	13.53	53%	13.15
**Neutral**	89%	12.61	82%	11.07
**Pride**	42%	27.22	36%	26.16
**Sadness**	79%	15.08	62%	16.81
**Surprise**	92%	9.75	70%	11.30

*Note*. M = Mean; SD = Standard Deviation.

**Fig 4:**
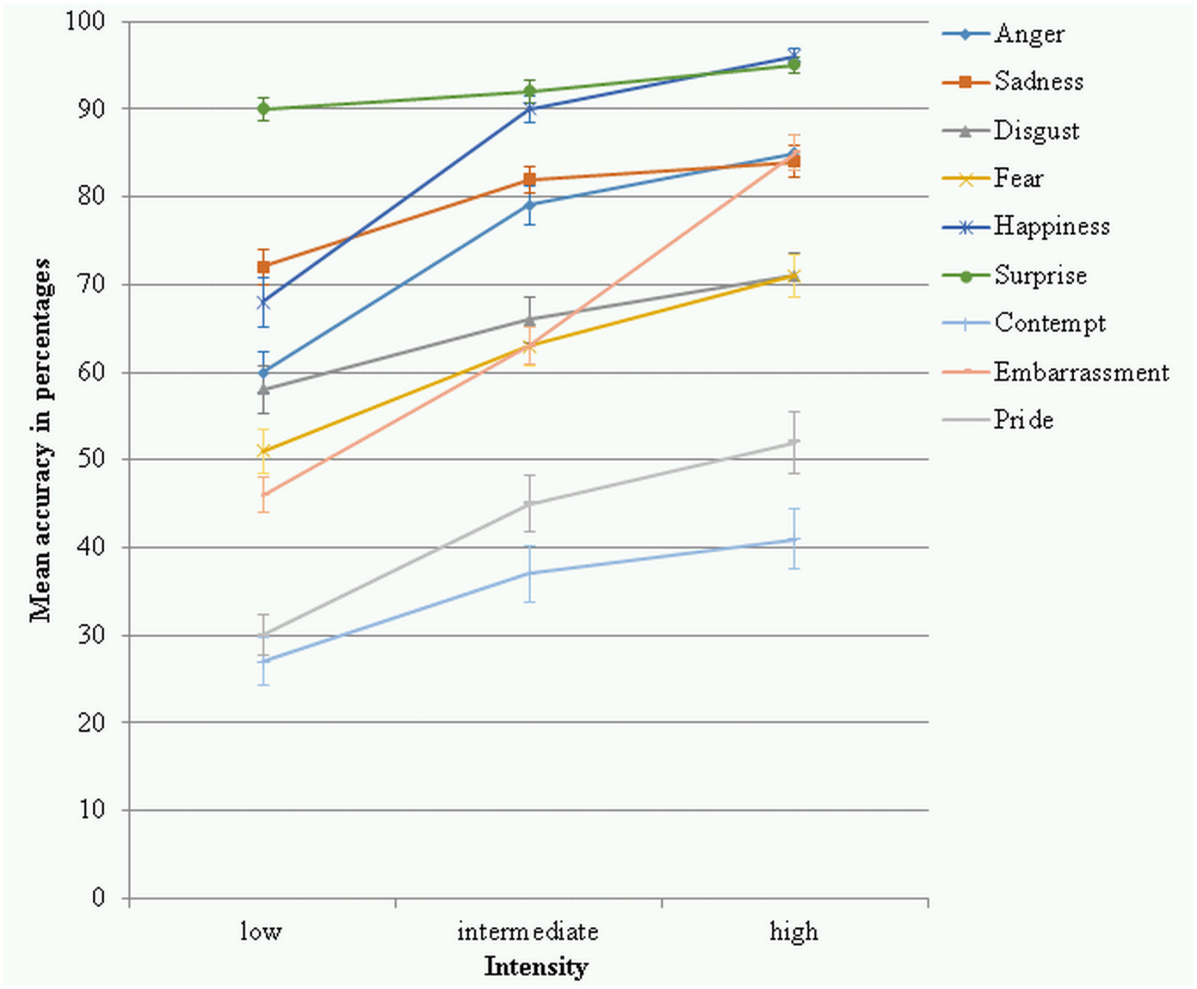
Raw hit rates in percentages for the 9 emotion categories at each of the 3 intensity levels. Error bars represent standard errors of the means.

The intensity x emotion interaction was significant (*F*(10.99, 999.93) = 20.14, *p* < .001, partial *η²* = .181, power = 1.000) (see Fig 4). Pairwise comparisons showed that the raw hit rates of the intensity levels within each emotion were significantly different from each other for most of the emotions (*p*’s < .037) except for sadness where the intermediate intensity was not significantly different from the high intensity (*p* = .154) and surprise where the low intensity was not significantly different from the intermediate intensity level (*p* = .103). Pairwise comparisons were conducted comparing the raw hit rates of the emotions to each other within each intensity level. Most emotions were significantly different from each other (*p*’s < .042). At low intensity anger was not significantly different from disgust (*p* = .709), as so contempt and pride (*p* = .411), embarrassment and fear (*p* = .095), and happiness and sadness (*p* = .174). At intermediate intensity anger and sadness were not significantly different from each other (*p* = .190), as so disgust and embarrassment (*p* = .399), disgust and fear (*p* = .364), embarrassment and fear (*p* = .950), and happiness and surprise (*p* = .210). At high intensity anger and embarrassment were not significantly different from each other (*p* = .840), as so disgust and fear (*p* = .979), embarrassment and sadness (*p* = .695), and happiness and surprise (*p* = .256). Table 2 shows the means and standard deviations of the raw hit rates for each emotion category at each intensity level.

**Table 2:** Raw Hit Rates (*H*) for the Emotion Categories by Intensity.

Emotion (*n* = 92)	*H* Means (Standard Deviations)		
	low	intermediate	high
**Anger**	60% (22.58)	79% (20.92)	85% (17.92)
**Sadness**	72% (18.83)	82% (14.42)	84% (17.33)
**Disgust**	58% (25.42)	66% (25.08)	71% (24.17)
**Fear**	51% (24.33)	63% (24.42)	71% (23.42)
**Happiness**	68% (26.75)	90% (14.67)	96% (7.67)
**Surprise**	90% (12.67)	92% (12.17)	95% (9.33)
**Contempt**	27% (17.75)	37% (31.58)	41% (32.42)
**Embarrassment**	46% (19.25)	63% (20.83)	85% (18.83)
**Pride**	30% (21.83)	45% (30.83)	52% (33.83)

One sample *t*-tests were conducted to test if the raw hit rates for each of the 27 categories were significantly different from chance level of responding (10%). One sample *t*-tests showed that with a Bonferroni-corrected *p* value of .002 all categories were recognised above chance (*t*(91)’s > 6.29, all *p*’s < .001).

#### DV 2: Unbiased hit rates

The *overall* accuracy of response (unbiased hit rates) for the 360 videos was 53% (*SD* = 11.28). The low intensity videos collapsed across emotions had an unbiased hit rate of 43% (*SD* = 11.51), 54% for intermediate intensity (*SD* = 12.00), and 63% for high intensity (*SD* = 12.54).

Most of the emotion categories were non-normally distributed with some left- and some right-skewed according to the histograms. Transformations did not normalise the data, but repeated measures ANOVA were conducted because it is robust to normality violations [65, 66]. A 3 (intensities) x 9 (emotions) repeated measures ANOVA was conducted with Greenhouse-Geisser adjustments of degrees of freedom due to violation of Sphericity. There was a significant main effect of *intensity* (*F*(1.84, 167.49) = 319.62, *p* < .001, partial *η²* = .778, power = 1.000) and pairwise comparisons showed that the intensity levels were all significantly different from each other (*p*’s < .001) (see Fig 2).

The main effect of *emotion* was significant (*F*(4.96, 451.29) = 61.49, *p* < .001, partial *η²* = .403, power = 1.000) (see Fig 3). Pairwise comparisons showed anger, disgust, embarrassment, fear, and happiness were not significantly different from each other (*p*’s > .172); all other categories were found significantly different from each other (*p*’s < .001). The means and standard deviations of the unbiased hit rates for the 9 *emotion* categories and neutral are presented in Table 1.

**Fig 5:**
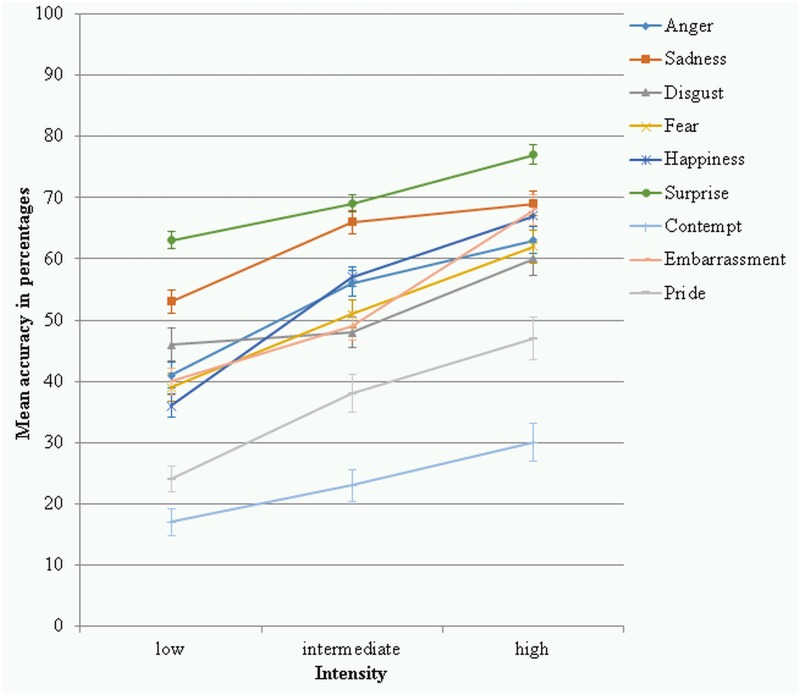
Unbiased hit rates in percentages for the 9 emotion categories at each of the 3 intensity levels. Error bars represent standard errors of the means.

The *intensity* x *emotion* interaction was significant (*F*(10.71, 974.81) = 11.14, *p* < .001, partial *η²* = .109, power = 1.000) (see Fig 5). Pairwise comparisons were conducted to examine the unbiased hit rates for each emotion for the three intensity levels. For most of the emotions significant differences were found (*p*’s < .014); only for disgust the accuracies at low and intermediate intensity were not significantly different (*p* = .414). Pairwise comparisons were conducted comparing the unbiased hit rates of the emotions to each other within each intensity level. Most emotions were significantly different from each other (*p*’s < .042). At low intensity anger was not significantly different from embarrassment (*p* = .705), fear (*p* = .590), and happiness (*p* = .086), as so embarrassment and fear (*p* = .885), embarrassment and happiness (*p* = .182), and fear and happiness (*p* = .287). At intermediate intensity anger was not significantly different from fear (*p* = .072) and happiness (*p* = .899), disgust was not significantly different from embarrassment (*p* = .660) and fear (*p* = .250), embarrassment was not significantly different from fear (*p* = .433), and sadness and surprise were not significantly different from each other at intermediate intensity (*p* = .114). At high intensity anger was not significantly different from embarrassment (*p* = .128), fear (*p* = .581), and happiness (*p* = .191), disgust was not significantly different from fear (*p* = .529), embarrassment was not significantly different from happiness (*p* = .543) and sadness (*p* = .851), as so fear and happiness (*p* = .083), and happiness and sadness (*p* = .384). Table 3 shows the descriptive statistics of the unbiased hit rates for each emotion at each intensity level.

**Table 3:** Unbiased Hit Rates (*Hu*) for the Emotion Categories by Intensity.

Emotion(*n* = 92)	*Hu* Means (Standard Deviations)		
	low	intermediate	high
**Anger**	41% (20.18)	56% (20.95)	63% (20.89)
**Sadness**	53% (18.05)	66% (18.71)	69% (19.00)
**Disgust**	46% (26.16)	48% (24.19)	60% (26.44)
**Fear**	39% (22.48)	51% (22.20)	62% (25.34)
**Happiness**	36% (18.38)	57% (15.40)	67% (16.30)
**Surprise**	63% (13.38)	69% (13.12)	77% (15.10)
**Contempt**	17% (21.93)	23% (24.40)	30% (29.69)
**Embarrassment**	40% (12.85)	49% (21.29)	68% (23.95)
**Pride**	24% (19.96)	38% (30.00)	47% (33.43)

One sample *t*-tests were conducted to test if the unbiased hit rates for each of the 27 categories were significantly different from chance level (10%) and showed that with a Bonferroni-corrected *p* value of .002 all categories except for contempt at low intensity (*t*(91) = 2.95, *p* = .004) were recognised above chance (*t*(91)’s > 4.95, all *p*’s < .001).

#### DV 3: Response times

Inspection of the Shapiro-Wilk statistics revealed the response time data for the intensities (correct trials only) were non-normally distributed (low: *S-W* = .92, *df* = 92, *p* < .001; intermediate: *S-W* = .93, *df* = 92, *p* < .001; high: *S-W* = .93, *df* = 99, *p* < .001) and therefore normalised using log transformation (low: *S-W* = .98, *df* = 92, *p* = .164; intermediate: *S-W* = .99, *df* = 92, *p* = .480; high: *S-W* = .99, *df* = 99, *p* = .528).

**Fig 6:**
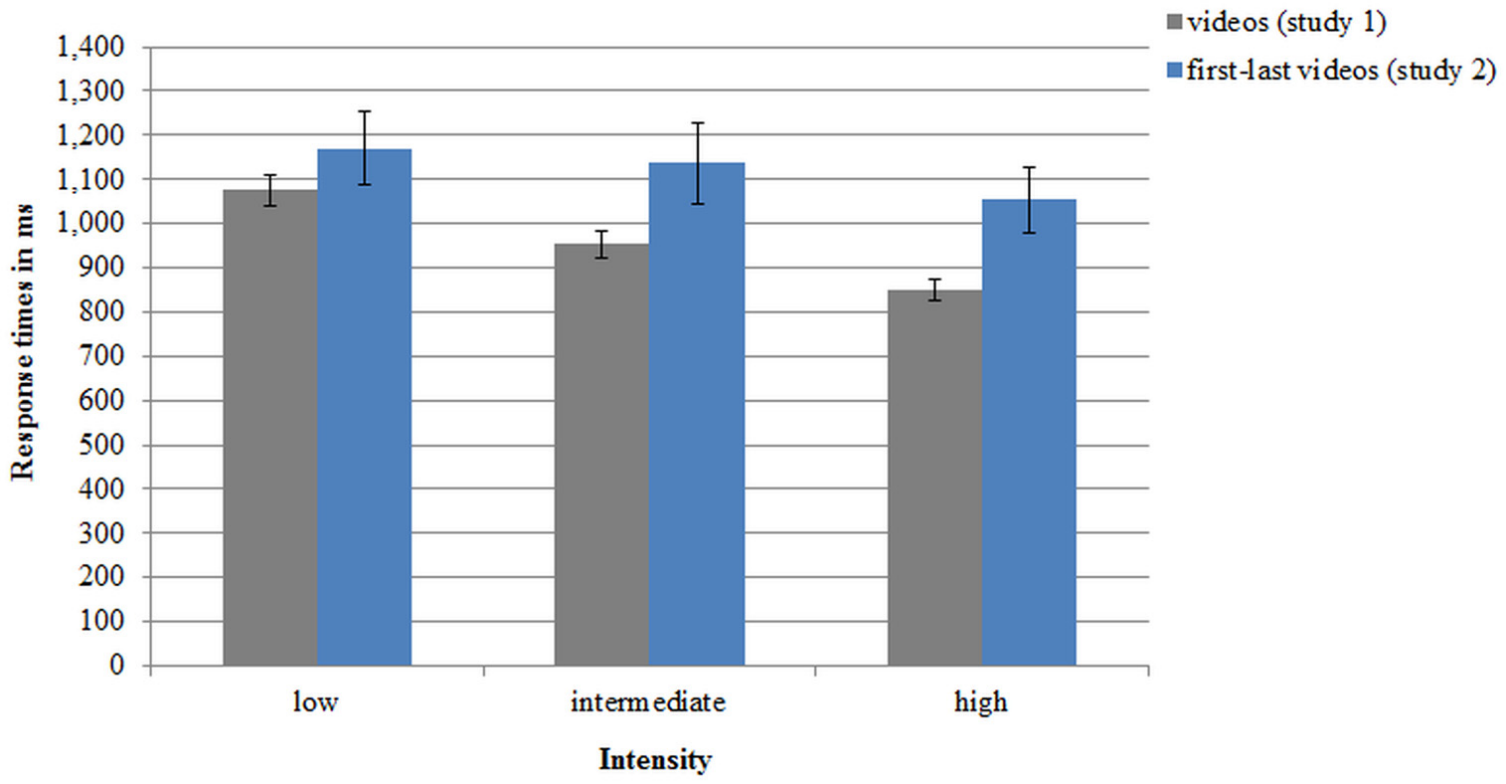
Response latencies (in ms) to the three intensity levels of the ADFES-BIV videos from study 1 and the first-last videos from study 2. Error bars represent standard errors of the means.

A repeated measures ANOVA for *intensity* with its three levels and revealed a main effect of *intensity* (*F*(2, 182) = 120.38, *p <* .001, partial *η²* = .569, power = 1.000). Pairwise comparisons showed, the intensities were all significantly different from each other (*p*’s < .001). It took the participants about 100ms longer to respond to low intensity (*M* = 1075ms, *SD* = 330) than to intermediate intensity (*M* = 953ms, *SD* = 296) and to intermediate intensity also about 100ms longer than to high intensity (*M* = 850ms, *SD* = 240) (see Fig 6).

**Fig 7:**
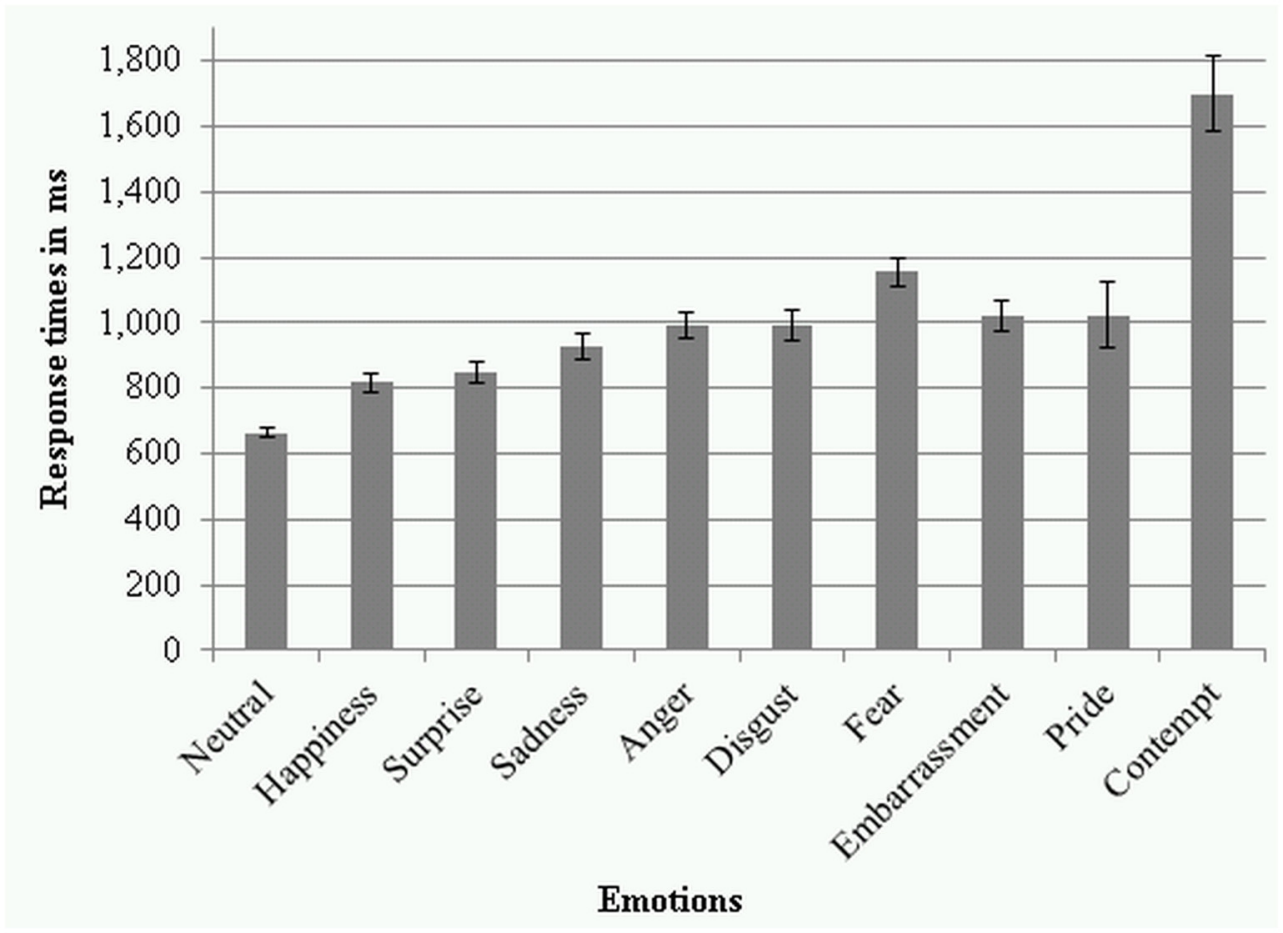
Response latencies to the ten emotion categories of the ADFES-BIV in ms. Error bars represent standard errors of the means.

The mean response times for each emotion category were calculated based on correct responses only: neutral (*M* = 664ms, *SD* = 145), happiness (*M* = 816ms, *SD* = 280), surprise (*M* = 849ms, *SD* = 285), sadness (*M* = 926ms, *SD* = 370), anger (*M* = 992ms, *SD* = 385), disgust (*M* = 994ms, *SD* = 451), fear (*M* = 1156ms, *SD* = 413), embarrassment (*M* = 1022ms, *SD* = 429), pride (*M* = 1023ms, *SD* = 937), contempt (*M* = 1700ms, *SD* = 1054) (see Fig 7).

### Discussion

Study 1 has shown, as hypothesised, that the mean accuracies for the emotion categories at each intensity level were well above chance for the 27 categories of the raw hit rates, as well as the unbiased hit rates with the exemption of the unbiased hit rate of contempt at low intensity. In line with the prediction, the emotion categories differed in accuracies and response latencies with emotions of high recognition also yielding fast responses and vice versa. Results also showed differences between levels of intensity of the expressions in both, the unbiased hit rates and raw hit rates, with the lowest mean accuracy for the low intensity expressions (raw: 56%, unbiased: 43%) compared to the intermediate intensity expressions (raw: 68%, unbiased: 54%), which were lower than the high intensity expressions (raw: 75%, unbiased: 63%). The current study further found that the intensity of facial expressions has an influence on response times, as significantly faster responses of about 100ms occurred between the intensities. Fastest responses were given to high intensity expressions and slowest responses to low intensity expressions, also in line with the prediction.

The differences in accuracies and response latencies across intensity levels can be explained by varying difficulty to recognise the expressions at varying intensities of expression. That is, facial emotional expressions of high intensity are easier to recognise than those of low intensity which reflects in higher accuracy and faster responses to high intensity expressions and lower accuracy and slower responses to low intensity expressions. Facial emotional expressions are harder to recognise at lower intensities because those expressions contain fewer cues that can be used for decoding.

However, there were differences between the intensities in display time of the emotional expressions seen by participants. In the low intensity videos of the ADFES-BIV the emotional expression was visible for less time than in the intermediate and high intensity videos, and the intermediate intensity videos had the expression displayed for less time than the high intensity videos. The resulting differences in processing time could be underlying the results, rather than the intensity of the facial expressions. To address this issue, versions of each video from the ADFES-BIV were created such that the last frame of the emotion was visible for exactly the same amount of display time across low, intermediate, and high intensity. Therefore, if the amount of time the expression was displayed across intensity levels was causing differences in accuracy rates and response latencies, these differences across intensity levels should be lost with this variation of the videos as the display times were equated. Instead, if it is the degree of intensity that is important rather than the amount of time the expression is displayed, then the same differences in accuracy rates and response latencies should be evident across the different intensity levels similar to Study 1.

## Study 2

Study 2 aimed to validate the results from study 1 that the intensity levels differ from each other in accuracy and response latencies by controlling for exposure time. A first-last approach was chosen for developing the control stimuli of the ADFES-BIV where the first and last frame of the videos are shown. Although this means that the progression and temporal characteristics of the individual expressions are lost, the perception of motion remains however due to the change from neutral to emotional facial expression. Since temporal characteristics are argued to be part of emotion representation [36] and therefore aid recognition [35, 68], the first-last approach leads to lower accuracy rates than complete dynamic sequences (see [34, 68]). Therefore, lower accuracies were expected for the control stimuli than for the ADFES-BIV, but with the same pattern of recognition and response times: highest accuracy rates and fastest responses to the high intensity videos, lowest accuracy and slowest responses to the low intensity videos.

### Method

#### Participants

Thirty individuals (15 females, 15 males) were recruited from the University of Bath community. Participants were PhD students (*n* = 16), Undergraduate students (*n* = 10), Masters students (*n* = 1), and staff (*n* = 3) and came from diverse departments. All participants had normal or corrected-to-normal vision. Participants were compensated with £5 or gained course credit (Psychology students) for participation. The age ranged from 18 to 60 years (*M* = 27.67, *SD* = 9.85). One person indicated having a diagnosis of an Anxiety Disorder. Since the data for this participant was comparable to the other participants, their data was included in the analysis. Due to a technical fault response time data were only available for 29 participants (-1 male).

#### Stimuli

From the 360 videos of the ADFES-BIV (Study 1) the first and last frame were extracted for each video. Video sequences were created with the first frame (neutral expression) being presented 13 times and then the last frame (emotional expression) for the same amount of repetitions with a speed of 25 frames/sec. It was chosen to apply the same characteristics to the control stimuli as to the ADFES-BIV videos, i.e. equal amount of frames (26) and speed of presentation (25 frames/sec) as well as starting with a neutral image and ending with an emotional expression. Thereby, highly standardised videos in regards to timings were created.

#### Procedure and task

The same procedure and task were applied as in Study 1. Raw hit rates and response times were the DVs. Ethical approval for this study was given by the University of Bath Psychology Ethics Committee and all participants gave written informed consent prior to participation.

### Results

#### DV 1: Raw hit rates

Raw hit rates were calculated for *intensity*. The overall accuracy of response for the 360 videos was 66% (*SD* = 8.72). For all low intensity videos taken together an accuracy of 51% was achieved (*SD* = 9.16), 66% for intermediate intensity (*SD* = 11.78), and 74% for high intensity (*SD* = 9.91). Inspection of the Shapiro-Wilk statistics revealed the raw hit rates data for the intensities were normally distributed (low: *S-W* = .98, *df* = 30, *p* = .698; intermediate: *S-W* = .94, *df* = 30, *p* = .115; high: *S-W* = .95, *df* = 30, *p* = .201). A repeated measures ANOVA with *intensity* as within-subject factor was conducted and a significant main effect of *intensity* was found (*F*(2,58) = 252.70, *p* < .001, partial *η²* = .897, power = 1.000) and pairwise comparisons showed the three intensity levels were all significantly different from each other (*p*’s < .001).

#### DV 2: Response times

Response times were calculated for *intensity* (correct trials only). Inspection of the Shapiro-Wilk statistics revealed the response time data for the intensities were non-normally distributed (low: *S-W* = .93, *df* = 29, *p* = .048; intermediate: *S-W* = .90, *df* = 29, *p* = .009; high: *S-W* = .93, *df* = 29, *p* = .062) and therefore normalised using log transformation (low: *S-W* = .99, *df* = 29, *p* = .994; intermediate: *S-W* = .97, *df* = 29, *p* = .656; high: *S-W* = .98, *df* = 29, *p* = .764).

A repeated measures ANOVA with *intensity* as within-subject factor was conducted to test if the intensities (correct trials only) differed significantly in response time. A significant main effect of *intensity* was found (*F*(2, 56) = 4.28, *p* = .019, partial *η²* = .133, power = .724). Pairwise comparisons showed that the responses occurred significantly faster for the high intensity level (*M* = 1054ms, *SD* = 399) than the intermediate intensity (*M* = 1137ms, *SD* = 491, *p* = .014). Responses did not occur significantly faster for the intermediate intensity than the low intensity level (*M* = 1171ms, *SD* = 452, *p* = .480). The responses to the high intensity level were significantly faster than to the low intensity level (*p* = .011) (see Fig 6).

### Discussion

In line with the prediction, the accuracy rates for the three intensity levels of expression in Study 2 were found to be significantly different from each other, with the low intensity having lower accuracy than intermediate intensity, which in turn had lower accuracy than the high intensity expressions. This rank order shows that the intensity of expression influences recognition independent from the display time, which was kept constant in this experiment. If the display time were the factor to modulate the accuracies for these videos, the intensity levels would not have been significantly different from each other, as the display time of emotional (and neutral) content was exactly the same for the different intensities. It can be concluded that even though the exposure time was kept constant, the low intensity category was still harder to correctly recognise than the intermediate one and the high intensity expressions were easiest to recognise. This demonstrates that it is the intensity of expression, and not display and processing time, which influences recognition accuracy from the videos.

A similar pattern of results to the accuracy rates was found for the response times. The mean response time for the low intensity stimuli was 117ms longer than for the high intensity stimuli, which is in line with the prediction. This means that even when the same processing time is allowed for the different intensities, responses occur slower for low intensity expressions than for high ones. Just as for accuracy, the expression intensity is of higher relevance for responding times than the display time.

## General Discussion

The main aim of the studies reported here was to validate a stimulus set of facial emotional expression videos of basic and complex emotions at varying intensities—the ADFES-BIV. The overall raw accuracy rate for facial emotion recognition of the ADFES-BIV videos was 69%, which is in line with other well validated and widely used video stimulus sets such as the MERT [47], which too had an overall accuracy rate of 69% in the video modality of the set. The overall accuracy based on unbiased hit rates was lower, as expected when correcting for biases, but still well above chance with 53%. With the studies reported here, the ADFES-BIV was successfully validated on the basis of raw and unbiased hit rates on all its level, i.e. intensity levels, emotion categories, and the emotions at each intensity level. Together, the results showed that this newly created video set of different intensities of emotional expressions of basic and complex emotions is a valid set of stimuli for use in emotion research.

### Validation of the intensity levels

One aim of the current research was to validate the three created intensity levels. As hypothesised, the rank order of the intensities was the same across the three dependent variables investigated with faster and more accurate responses to higher intensities than low intensity expressions, meaning the low intensity expressions are hardest to recognise and the high intensity expressions are easiest to recognise. The linear increase of approximately 10% in accuracy from low intensity expressions (raw: 56%, unbiased: 43%) to intermediate intensity expressions (raw: 68%, unbiased: 54%) to high intensity expressions (raw: 75%, unbiased: 63%) of the ADFES-BIV is in line with previous research from morphed static stimuli looking at varying intensities where accuracy was linearly associated with physical expression intensity (see [44]) and has been shown for the first time from dynamic stimuli, i.e. video recordings. The lower response times in higher intensities support the notion that responding occurs more rapidly the easier a stimulus is to recognise [28].

The intensity of the muscle contractions underlying the facial emotional expressions and the amount of facial action units activated constitute the determining factor regarding ease of recognition. That is, the higher the intensity of muscle contraction, the more apparent the facial features that form the emotional facial expressions, which aligns with the ratings of perceived intensity within this study (see also [60]) and facilitates recognition in both accuracy and response time (see also [53]) and explains the found differences between the intensities within the current research. At emotion onset not all facial action units are activated (e.g. [44, 69]) and the ones that are activated have not reached their maximum yet. That subtler emotional expressions are less well recognised than intense expressions even though we experience them frequently in social interactions can be explained evolutionary. With facial emotional expressions serving the communicative function of signalling threat (among others) [70], it can be assumed that the level of threat correlates with the intensity of the expression, i.e. the more intense the expression, the more important to recognise the expression. A reason for why subtle emotional facial expressions are harder to recognise than more intense expressions can also be found in the intention of the encoder. In some situations people mask their feelings based on social norms influenced by culture, context, and personality etc. [71] leading to only subtle expressions (intentionally) hard to recognise. In this case, it can actually be beneficial in the interaction to not perceive and recognise the emotion. In this context, it would be interesting to examine if highly empathic people are better than low empathic people at recognising subtle emotional expressions; a question that could be investigated with the ADFES-BIV.

Next to the intensity of expression, the found differences in accuracy and response latencies between the intensity levels from the ADFES-BIV could also be explained by the varying amount of display time of emotional content between the intensities in the videos. This was tested in Study 2 with controlled exposition time and results showed the same pattern of accuracy and response latencies for the different intensities as in Study 1. In the ADFES-BIV the accuracy increased by 12% from the low to intermediate category and by 7% for the intermediate to high intensity. Similarly, in Study 2 the accuracy increased by 15% from low to intermediate intensity, and by 8% from intermediate to high intensity. In both studies responses occurred faster to high intensity expressions than low intensity, although in Study 2 the differences between the intensities were smaller from low to intermediate than from intermediate to high, whereas in Study 1 the differences were consistent. This demonstrates that the response latencies were only a little influenced by display time and motion was more influential; accuracy was not influenced by the varying display times. Due to the very similar results from Study 1 and 2, it can be concluded that the varying exposure times to emotional content have little influence on recognition for the present stimulus set, i.e. the ADFES-BIV accuracies were not confounded by exposure time. Instead, the intensity of expression determined the difficulty of recognition. Results support the distinction of low, intermediate, and high intensity, since the categories were found to differ from each other in accuracies and response latencies and validates the intensity levels of the ADFES-BIV as distinct categories.

### Validation of the emotion categories

Next to the intensity levels, the aim of the current research was to validate the ADFES-BIV on its emotion categories. The raw hit rates of the emotion categories yielded recognition significantly above chance level of responding, in line with the prediction. The rank order of recognition based on the raw hit rates found from the ADFES-BIV is in line with the existing literature where surprise and happiness are usually reported as the emotions of highest recognisability (e.g. [26]) and are therefore deemed easiest to recognise. The high recognition of happiness can be explained by the fact the facial expression of happiness is very distinct from all other basic emotions. The smile is a very visually salient feature and most important, maybe even the only necessity, for happiness recognition [72]. Of the basic emotions fear is usually the category of lowest recognition (e.g. [27]) and therewith hardest to recognise, which has also been found with the ADFES-BIV. Also in line with existing research is that the recognition rates of the complex emotions included in the ADFES-BIV led to lower recognition than the basic emotions (in line with the original ADFES [33]) with contempt having been the hardest to recognise shown by the lowest recognition rates (see also [73]). An explanation for this particular rank order comes from research correlating the familiarity with particular emotions based on the encounters in daily life with recognition and has found a strong positive relationship [25]. It seems that we are better at recognising the emotions that we encounter most frequently. An additional explanation comes from the evolutionary perspective where it is claimed that we are innately set up to recognise the basic emotions, as this is functional for survival [5,19]. Overall, the results show that the emotion categories of the ADFES-BIV have been successfully validated on the basis of raw hit rates.

Also the unbiased hit rates of the emotion categories were significantly above chance level of responding for all categories as predicted. Since the unbiased hit rates are corrected for confusions, their hit rates were lower than the raw hit rates. The rank order of recognition changed slightly. Even though to a lesser degree than the raw hit rates, surprise was still the emotion best recognised and contempt least. Happiness was no different in its unbiased hit rates than anger, disgust, fear, and embarrassment. These findings can be explained by confusions that predominantly happen for certain emotions, such of high featural overlap. For example, happiness and pride share the feature of a smile which is why participants can confuse pride expressions with happiness (see also [58]), which lowers the unbiased hit rates for happiness. The lower unbiased hit rates for surprise can be explained by confusions of fear with surprise and the lower unbiased hit rates of anger are rooted in the confusions of disgust with anger. These confusions are typical and have been previously reported (e.g. [37, 58, 74]). (The confusion matrix can be retrieved from the supporting information; S1 File). Due to the morphological similarity of those emotion pairs, especially early in the unfolding expressions, it has been suggested that those emotion pairs constitute basic emotions themselves leaving us with four and not six basic emotions [69]. Together, the recognition rates show that the emotion categories of the ADFES-BIV have been successfully validated on the basis of unbiased hit rates.

Contempt was the emotion that resulted in the lowest accuracies on both raw and unbiased hit rates. Given that contempt is suggested to constitute a basic emotion [75], it should be more distinct and better recognised. However, the low contempt recognition rate in the current study is in line with the literature based on English-speaking subjects (e.g. [58, 76, 77]). The original ADFES was validated on a Dutch-speaking sample and could explain their better performance on contempt recognition, because there is reason for the assumption that the concept ‘contempt’ entails slightly varying meaning between languages affecting recognition rates achieved by native English speakers [76]. Application of the ADFES-BIV in other languages than English with respective translations of the emotion terms would shed light on whether it is the category term ‘contempt’ that is problematic, or the videos are not properly representing contempt. It is possible that the prototypical display of contempt was not truly captured by the encoders in the videos or a different label (e.g. disapproval) would represent better the facial expression, which could be tested empirically within a freely labelling task.

### Validation of the emotion categories at each intensity level

A further aim was to validate the ADFES-BIV emotion categories at each intensity level. In line with the prediction, the emotion categories at each intensity level also yielded raw hit rates significantly above chance level of responding. For the unbiased hit rates contempt at low intensity was found to be difficult to recognise, shown by accuracies not significantly different from chance level of responding. However, an increased intensity of contempt facial expressions (i.e. intermediate) has led to accuracies significantly above chance level of responding in the unbiased hit rates, and the raw hit rates for contempt at low intensity were above chance level. The results show that the emotion categories at each level of intensity of the ADFES-BIV have been successfully validated on basis of raw hit rates and unbiased hit rates (except for contempt at low intensity). Overall, the results show that the ADFES-BIV is suitable for application in facial emotion recognition investigations.

### Influence of intensity on emotion recognition

Another aim of the current research was to investigate the influence of intensity on recognition based on the raw hit rates. Against the prediction, even the easy to recognise emotions were less well recognised at low intensity than at high intensity, shown by significant differences between those intensities. Happiness and surprise were best recognised across the intensities compared to the other emotions and had comparable high accuracies to each other at intermediate and high intensity, but significantly lower accuracies at low intensity were found for happiness compared to surprise. It seems that happiness is more ambiguous at low intensity than surprise. The importance of a salient smile in happiness for recognition [78] explains the lower recognition at low intensity where only a little smile is visible in the stimuli.

The greater emotional information available at intermediate compared to low intensity expression of surprise did not increase the raw hit rate significantly. Accordingly, with an accuracy of 90% at low intensity surprise constitutes a very clear expression. The very good recognition even at low intensity might be rooted in the importance of surprise recognition. Surprise has been suggested to be comparable to a startle response rather than an emotion as it generally precedes other emotions in the case of an unexpected event and can turn into for example happiness or fear [79], giving surprise substantial signalling/alerting character and making it necessary to be recognised easily to prepare for action. Alternatively, surprise could therefore be deemed the most basic of the basic emotions, making the recognition less dependent on expression intensity.

After the raw hit rates have been corrected for confusions, surprise remained the best recognised across the intensities and contempt the least. However, the remaining emotions were in closer proximity to each other. Intensity of expression seemed to have a bigger influence on the unbiased hit rates than the raw hit rates, since (except for disgust) all hit rates increased from low to intermediate to high intensity. With more emotional cues available, fewer confusions occur, which increased the unbiased hit rates. At low intensity, more confusions of disgust and anger as well as fear and surprise are to be expected, as at the onset of the expressions the shared feature is most salient and discriminative information available later in the sequence of expression [69]. It can be concluded that intensity of the facial expression is important for the recognition of all emotions.

### Connection of accuracy and response time

Another aim of the current research was to examine whether or not the rank order of recognition reflected in response latencies. Indeed, the emotion categories that were best recognised were also the categories with shortest response latencies (i.e. happiness, surprise) and the emotion categories with the lowest accuracy rates were the categories for which responding was slowest (i.e. fear, contempt), in line with reports by Tracy and Robins [58]. Though, the varying response latencies are interesting, since only correct answer trials were considered in the response latencies calculations. One explanation for the varying response latencies is that even though participants gave the correct answer, they were uncertain about their answer, which slowed down their responding due to hesitation, which could be assessed by confidence ratings in future research. It is also possible that more explicit recognition strategies requiring more time were applied for the emotions that are harder to recognise. Another possibility is that emotions containing very salient features early on in the expression are recognised earlier [78] or that some emotions are processed differently in the brain, through different pathways or brain regions resulting in varying processing and therewith response times. It has been proposed [30] and shown that different emotions are processed differently neurally [80,81]; how this relates to response latencies is a question for future research.

### Advantages of video stimuli of facial emotional expressions

When conducting a facial emotion recognition experiment the impact of the nature of the stimuli should not get neglected. The accuracies for the intensities measured in Study 2 were consistently lower than in Study 1 and response times were consistently longer than in Study 1. Although the stimuli applied in Study 2 provided a perception of change—the change from neutral to the emotional expression—the emotion-specific temporal characteristics containing important information for decoding [82,83] were stripped. This explains the lower accuracy rates found for the stimuli in Study 2 than from the truly dynamic videos. This demonstrated that motion/dynamics are important for recognition [34,68], especially for subtle expressions, as the perception of change provides further information useful for decoding [9]. That motion facilitates facial emotion processing is supported by the findings of the current research, since the duration of emotional content visible was kept constant in Study 2. Even though participants here saw the low intensity expressions for longer than in Study 1, they took about 100ms longer to respond. If exposure time had an influence on response time, the response times in Study 2 should have been shorter than in Study 1, at least for the low intensity category. As much as dynamic facial expressions, i.e. motion, facilitate recognition (e.g. [35]) they facilitate response time; probably by facilitating the visual processing of the emotional expression per se. Using brain imaging Yoshikawa and Sato [84] found dynamic stimuli to elicit perceptual enhancement compared to static facial expressions which was evident in greater brain activity in relevant emotion processing regions of the brain, and they concluded that stimulus dynamics help to facilitate brain processing and, thus, the resulting face processing (see also [85–87]).

As has been shown with the current research, motion facilitates emotion processing. However, regarding the dynamics, there are a number of things to consider, especially when applying/developing morphed dynamic stimuli. Hara and Kobayashi (as cited by [45]) analysed the time between emotion onset and apex of the six basic emotional expressions from videos and found a range from 330ms to 1,400ms for the fastest to slowest moving emotion (surprise and anger vs. sadness, respectively). These temporal characteristics seem to be embedded in our representations of emotions. For example, Sato and Yoshikawa [36] used morphed sequences to show that the perceived naturalness of an emotional facial expression was dependent on the speed of which it was displayed developing. The speed perceived most natural was found to differ between emotions with surprise being considered most natural at a fast pace and sadness as such when moving slowly—matching the measured speed of expression (e.g., by [88]). If stimuli alterations result in a for the emotion unnatural speed, this not only lowers ecological validity it can further lead to diminished recognition [35, 68]. It can be assumed that the internal representations of facial emotional expressions we use for recognition of such do include both emotion-specific facial features and their temporal characteristics [35]. The latter is not captured in static stimuli and generally not in morphed sequences where the same temporal characteristics are typically applied to all emotional expressions when facial emotion recognition is investigated.

With respect to the visible facial features, it is possible that the onsets of facial action units within an emotional expression vary [69]. For example in happiness, at a very low intensity of expression, the wrinkling around the eyes is not visible, but the smile is [44], which would mean that the mouth corners are pulled up before the wrinkling around the eyes occurs. True (un-manipulated) video recordings capture this, as timings are preserved, whereas morphed sequences present simultaneous appearance changes of the facial features.

Another point to consider is that there is inter-individual variability that can be found in the speed of emotional expression [88] in facial expressions. Pollick et al. [88] used 3-D point light displays of posed expressions (anger, happiness, sadness, and surprise) recorded to video and assessed the time of expression from onset to apex of four encoders. They found the speed of facial expressions to range by 466ms between the slowest and fastest encoder across the expressions included. Interestingly, this range was bigger than the one between the slowest and fastest emotion, which was 208ms. Again, those variations are captured by true video recordings, but not in morphed sequences, demonstrating the benefit of using video recordings.

### Limitations

The varying durations of emotional content visible between the intensities of the ADFES-BIV could be seen as limitation in terms of standardisation. However, they could also be interpreted as a further indicator of ecological validity, as the point of onset always precedes the reaching of maximum expression intensity. It is virtually impossible to create stimuli of facial expression at varying intensities and have the emotional content visible for the exact same duration between the intensities without manipulating the expression dynamics. Given the anatomical underpinnings of muscular movements, expressing low intensity facial emotional expressions should take less time than high intensity ones, since a more intense muscle contraction should take more time. Except if it was the case that we speed up our facial expression when expressing full intensity and slow down for subtle expressions which seems unlikely, but research on that has yet to be undertaken. Further support comes from the DaFEx video database [56] where professional actors were specifically instructed to express low, intermediate, and high intensity emotional facial expressions leading to the videos in the low condition being shorter in lengths than the intermediate ones and the latter shorter than the high intensity videos, just as in the current study. It can therefore be concluded that even though the current videos were edited, variations in timings between intensities resemble a natural occurrence for posed expressions—the intended purpose of the stimuli.

A limitation of the stimuli is that the videos are edited to varying intensities of expression rather than having encoders instructed to express low, intermediate, and high emotional expressions. However, regarding the facial features this does not propose a strong limitation, since muscles can only do specific movements, which are catalogued in the FACS [15]; it is only the intensity of muscle contraction that changes. For example, when making a sad facial expression as intense as possible, it always starts out as a subtle expression increasing to full intensity. Hence, it is legitimate to extract sequences and still be able to claim to have varying intensity levels. Yet, future research could instruct encoders to pose varying intensities and FACS code them to verify the present results.

The approach taken to create the stimuli within the current research led to a further limitation. That is, offsets of the facial expressions of emotions were not displayed within the videos, which is obviously unlike what we encounter in everyday social interactions. It would be necessary for future research to instruct encoders to portray varying intensities of facial expression while recording them, as suggested above, and document onsets, the duration of the emotional display, as well as offsets of the facial emotional expressions.

To increase ecological validity even further than including the whole range of the expressions from onset to offset, future research should also aim to produce videos of varying intensity portraying individuals expressing emotions facially that have been elicited in those individuals, i.e. truly felt emotions, to further increase ecological validity and tackle some of the issues raised in this discussion. Subjective ratings alongside FACS coding would be necessary to assure the elicited emotions equal the target emotion.

### Potential Applications for the ADFES-BIV

A variety of application options exist for the ADFES-BIV. The ADFES-BIV could find application in multimodal emotion recognition experiments, since emotion recognition is a multisensory process (e.g. [89]). As in social interactions next to visual emotional information usually also auditory emotional and contextual information is present [90] the ADFES-BIV could for example get combined with the Montreal Affective Voices set [91]. Where multisensory emotion recognition has been investigated including high intensity facial expressions [89], the current stimulus set allows for extension to subtle emotional expressions. The ADFES-BIV could also be applied to investigate group difference between clinical samples and controls in facial emotion recognition. For example, an unpublished pilot study has shown suitability for the application of the ADFES-BIV as an emotion recognition task in high-functioning autism. Future research could further apply the ADFES-BIV in neuroscientific research. For example, since most of the research is conducted using full intensity facial expressions research questions open to answer are whether the intensity of the observed expression reflects in the intensity of the resulting brain activity or if there even is a negative correlations, as subtler expressions are harder to decode, more neural processing is needed. As suggested by an anonymous reviewer, the ADFES-BIV could also be of interest for research on pre-attentive or non-conscious emotion perception. Facial emotional expressions of varying intensity could be used instead of altering exposure time or masking to investigate the limits of non-conscious emotion processing, both in healthy and brain damaged individuals. For example, such stimuli could find application in research on limited attention and awareness (e.g. [92, 93]).

## Conclusion

The ADFES-BIV, a video-based stimulus set of facial emotional expressions with different intensities, has been developed and validated, allowing for versatile applications in emotion research. This stimulus set can test a variety of emotions and intensity levels to investigate group differences or physiological/neurological responses to emotional face stimuli that are of interest in healthy and clinical samples. The ADFES-BIV can be retrieved free of charge for research purposes from the corresponding author. The first-last videos or the underlying static images can get retrieved upon request as well.

## Supporting Information

S1 Data Data from the study on the judgements of intensity of the ADFES-BIV videos. (XLSX)

S2 Data Accuracy of response data from study 1. (XLSX)

S3 Data Response times data from study 1. (XLSX)

S4 Data Accuracy of response data from study 2. (XLSX)

S5 Data Response times data from study 2. (XLSX)

S1 File Confusion Matrix for the Emotion Categories in Percentages. The diagonal shows the correct identifications (marked green). The percentages above and below the diagonal show the confusions of a target emotion with another category with values marked as red confusions greater than chance level (10%).
(TIF)
