# Correction: Validation of the Amsterdam Dynamic Facial Expression Set – Bath Intensity Variations (ADFES-BIV): A Set of Videos Expressing Low, Intermediate, and High Intensity Emotions

**DOI:** 10.1371/journal.pone.0168891

**Published:** 2016-12-15

**Authors:** Tanja S. H. Wingenbach, Chris Ashwin, Mark Brosnan

The email address of the corresponding author, Tanja S. H. Wingenbach is incorrect. The correct email address to reach the corresponding author is tanja.wingenbach@bath.edu.

The data availability statement is also incorrect. Please see the correct Data Availability Statement here: Some restrictions to data apply. Due to legal restrictions, the ADFES-BIV video set must be made available upon request to the corresponding author, Dr. Tanja Wingenbach (tanja.wingenbach@bath.edu). All other data are available within the paper and supporting information files.
