# Supplementary figures and images for: Validation of the Amsterdam Dynamic Facial Expression Set – Bath Intensity Variations (ADFES-BIV): A Set of Videos Expressing Low, Intermediate, and High Intensity Emotions
Source: PLoS One. 2016 Jan 19;11(1):e0147112. doi: 10.1371/journal.pone.0147112 (PMC4718603; doi:10.1371/journal.pone.0147112)

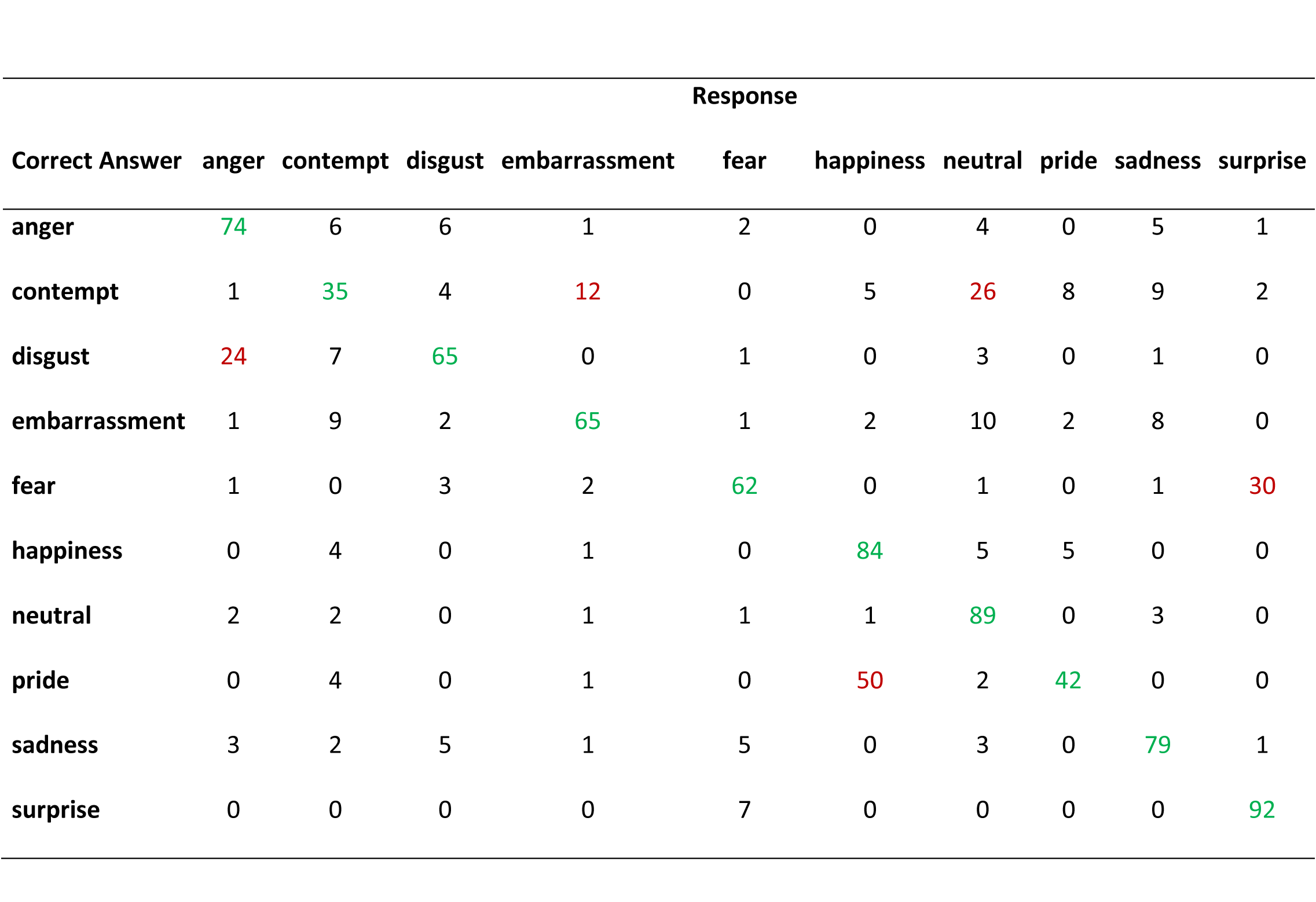

Supplement: S1 File — The diagonal shows the correct identifications (marked green). The percentages above and below the diagonal show the confusions of a target emotion with another category with values marked as red confusions greater than chance level (10%). (TIF) [file pone.0147112.s006.tif]
